# Temperature-Dependent Deformation Mechanisms in Ti65 Alloy: An In Situ Tensile Study

**DOI:** 10.3390/ma18143270

**Published:** 2025-07-11

**Authors:** Haitao Li, Chenxu Li, Dongmei Chen, Yujing Liu, Zibo Zhao, Bohua Zhang, Meng Qi, Jianrong Liu, Qingjiang Wang

**Affiliations:** 1 Shenyang Aircraft Design and Research Institute, Aviation Industry Corporation of China, Shenyang 110035, China; leastaony@163.com (H.L.); chendongmei2025@163.com (D.C.); 2 Yuhua Institute of Advanced Materials, Baoji Xigong Titanium Alloy Products Co., Ltd., Baoji 721300, China; l786307001@163.com (C.L.); bohua_zhang@163.com (B.Z.); qimengllm@163.com (M.Q.); 3 Institute of Metal Research, Chinese Academy of Sciences, Shenyang 110016, China; jrliu@imr.ac.cn (J.L.); qjwang@imr.ac.cn (Q.W.)

**Keywords:** Ti65 alloy, plastic deformation, in situ tensile test, high-temperature deformation, molecular dynamics simulations

## Abstract

Understanding the relationship between deformation behavior and mechanisms at elevated temperatures is of great significance for applications of high-temperature titanium alloys. This study systematically investigates the plastic deformation behavior of Ti65 alloy under both room-temperature and high-temperature conditions through in situ tensile testing, combined with slip trace analysis, crystal orientation analysis, and geometrical compatibility factor evaluation. TEM observations and molecular dynamics simulations reveal that plastic deformation is predominantly accommodated by basal and prismatic slip systems with minimal pyramidal slip contribution at room temperature. However, elevated temperatures significantly promote pyramidal <a> and <c+a> slip due to thermal activation. This transition stems from a shift in deformation mechanisms: while room-temperature deformation relies on multi-slip and grain rotation to accommodate strain, high-temperature deformation is governed by efficient slip transfer across grain boundaries enabled by enhanced geometrical compatibility. Consistent with this, thermal activation at elevated temperatures reduces the critical resolved shear stress (CRSS), preferentially activating 1/3<11–23> dislocations and thereby substantially improving plastic deformation capability. These findings provide critical insights into the temperature-dependent deformation mechanisms of Ti65 alloy, offering valuable guidance for performance optimization in high-temperature applications.

## 1. Introduction

High-temperature titanium alloys are widely valued for their exceptional specific strength, corrosion resistance, and thermal stability, exhibiting strong potential for applications in aerospace, energy, and chemical industries. The Ti65 alloy is a near-alpha high-temperature titanium alloy with a nominal composition of Ti-5.8Al-4.0Sn-3.5Zr-0.5Mo-0.4Si-0.3Nb-2.0Ta-0.8W-0.06C [1]. It currently holds the highest service temperature among titanium alloys and can operate stably at 650 °C for extended periods. Due to its excellent high-temperature strength, creep resistance, and oxidation resistance, it has emerged as one of the candidate materials for critical components such as compressor discs and blades in aerospace engines [1]. With the continuous improvement in the thrust-to-weight ratio of aerospace engines and the efficiency of gas turbines, higher demands have been placed on the heat resistance and mechanical properties of Ti65 alloy [2,3]. There are significant differences in the mechanical properties of Ti65 alloy at room temperature and elevated temperatures, primarily due to variations in plastic deformation mechanisms. A thorough investigation of the deformation mechanisms under different temperature conditions is of great significance for performance enhancement of high-temperature titanium alloys [4,5].

Currently, research on the plastic deformation mechanisms of high-temperature titanium alloys primarily focuses on three aspects: the critical resolved shear stress ratio [6,7,8], the activation of slip systems [9], and the twinning deformation [10]. For instance, Meng et al. [9] employed in situ electron backscatter diffraction (EBSD) to analyze microstructural evolution and slip activation within α colonies during the tensile deformation of Ti-6Al-4V alloy. Their results demonstrated that, during room-temperature in situ tensile testing, slip could propagate across the boundaries of parallel α lamellae with similar orientations as strain increased. Additionally, grain orientation rotation, driven by slip system activation, was observed during plastic deformation. Long et al. [11] investigated the microstructural evolution of Ti-6242S alloy during compression within the α+β phase field. Their findings revealed that the lamellar regions of α grains rotated to alternative orientations to enhance texture uniformity in the bimodal microstructure, thereby improving the mechanical properties of near-α Ti-6242S forgings. These statistically supported room-temperature findings provide critical insights into the plastic deformation mechanisms of high-temperature titanium alloys [12], particularly regarding slip behavior, crystallographic orientation evolution, and their influence on mechanical properties. However, the deformation mechanisms under high-temperature tensile conditions differ significantly from those at room temperature [13]. Research on high-temperature plastic deformation mechanisms remains limited and has predominantly examined other alloy systems. For example, Zhang et al. [14] combined in situ scanning electron microscopy (SEM) and EBSD to study the tensile mechanical properties, microstructure, orientation, and grain boundary evolution of Inconel 740H at 650 °C. Their work showed that grains rotated into favorable orientations to accommodate intergranular deformation and maintain matrix continuity. Similarly, the high-temperature tensile deformation behavior of cast GW103 alloy was investigated using in situ SEM and EBSD-based slip trace analysis to identify active slip systems [15]. The results indicated that basal slip was the predominant deformation mode across all tested conditions, while non-basal slip activation was temperature-dependent. Slip transmission between adjacent grains occurred during low-temperature testing, whereas grain boundary migration became prevalent at elevated temperatures, suggesting contributions from both dislocation slip and grain boundary sliding. Although these studies offer valuable perspectives on high-temperature deformation mechanisms, the synergistic interplay of multiple mechanisms remains insufficiently explored. Notably, the in situ plastic deformation behavior of Ti65 alloy under high-temperature conditions constitutes an understudied area. The complexity of high-temperature deformation poses a fundamental scientific challenge hindering this alloy’s further development and application.

In situ SEM and EBSD are highly effective in tracking the morphology and crystallographic orientation evolution [16,17]. In situ SEM enables dynamic observation of microstructural changes during tensile deformation, while interrupted testing allows for the characterization of slip activity, grain evolution, and microcrack initiation. This study integrates in situ SEM and EBSD to analyze the tensile deformation behavior of Ti65 alloy at room temperature and 700 °C. Through combined slip trace analysis, crystal orientation characterization, geometrical compatibility factor evaluation, along with TEM observations and molecular dynamics simulations, this study provides a comprehensive discussion on the tensile deformation mechanisms of Ti65 alloy. Elucidating these mechanisms is crucial for understanding its high-temperature mechanical behavior, optimizing thermomechanical processing, and expanding its engineering applicability.

## 2. Materials and Methods

The as-received material is a Ti65 bar forged in the α+β phase field with a diameter of 300 mm. Rectangular dog-bone-shaped tensile specimens with 13 × 5 × 1 mm^3^ gauge geometry were sectioned by electrical discharge machining (EDM) and ground on a series of SiC abrasive papers before being fine-polished with colloidal SiO_2_. Tensile testing was performed on an EDU4503 electronic universal testing machine (Kaiple, Changsha, China) at a strain rate of 2.8 × 10^−3^ s^−1^. Tests were conducted at both room temperature and 700 °C, with specimens heated at a rate of 10 °C/min. The electronic universal testing machine is equipped with a 10,000 N capacity force sensor (accuracy class: 0.1% FS) and a temperature sensor with a maximum measurement range of 1200 °C. Strain measurements in the gauge section were obtained using a video extensometer (Kaiple, Changsha, China). Each test condition was replicated three times to ensure result reliability.

For in situ testing, rectangular dog bone-shaped tensile specimens with 50 × 10 × 1.0 mm^3^ gauge geometry were sectioned by EDM, specimens were sequentially polished with progressively finer grit sandpaper before electropolishing at −30 °C in an electrolyte solution containing 5% perchloric acid, 35% n-butanol, and 60% methanol. In situ tensile experiments were performed inside a Thermo Fisher Apreo 2C field-emission scanning electron microscope (Thermoscientific, Prague, Czech Republic) equipped with a Zeptools PicoFemto in situ high-temperature tensile stage (Zeptools, Beijing, China), enabling dynamic observation of the deformation process at a constant displacement rate of 2 μm/s. The in situ high-temperature tensile stage is equipped with a 5000 N capacity force sensor (accuracy class: 0.05% FS) and a temperature sensor with a maximum measurement range of 1000 °C. EBSD analysis was conducted using a QUANTAX EBSD e-Flash fs/XFlash 7T detector (Bruker nano Gmbh, Berlin, Germany) with acquisition parameters set to 1 μm step size and 20 kV acceleration voltage. The thin foils for the STEM and weak-beam dark field observation were prepared using focused ion beam (FIB) milling (dual-beam FEI Helios 5 PFIB, Thermoscientific, Prague, Czech Republic). An FEI Tecnai F30 TEM (Thermoscientific, Prague, Czech Republic) operated at a voltage of 300 kV was used to evaluate the slip modes and microstructural evolution beneath the treated surface of the Ti65 alloy. All EBSD data were analyzed using the AZtecCrystal software 2.1. All TEM data were analyzed using the DigitalMicrograph software 3.53.

The MD simulations in this work were performed using the Large-Scale Atomic/Molecular Massively Parallel Simulator (LAMMPS 3 Mar 2020) software package. The polycrystalline model was constructed using the ATOMSK Version beta-0.13.1 software [17]. Periodic boundary conditions were applied in all three directions. The simulation box dimensions were set to 100 Å in the x, y, and z directions, containing 20 grains with different orientations, totaling approximately 50,000 atoms. This configuration ensures a balance between computational efficiency and accuracy.

The simulation setup was implemented as follows: firstly, the initial configuration of the polycrystalline model was minimized using the conjugate gradient (CG) method to eliminate non-physical atomic overlaps and high-energy states. The convergence thresholds for energy and force are both set to 1 × 10^−15^. After energy minimization, the system is equilibrated for 20 ps at the target temperatures of 273 K and 973 K under isothermal–isobaric (NPT) ensemble conditions to achieve thermodynamic equilibrium, with zero pressure maintained in the y and z directions. Subsequently, uniaxial tensile deformation is applied along the x direction at a constant strain rate of 1 × 10^9^ s^−1^, while maintaining zero stress conditions in the y and z directions. OVITO-3.12.4-x86_64 software is used for post-processing and visualization of simulation data. The dislocation extraction algorithm (DXA) is employed to characterize the evolution of dislocation structures throughout the deformation process, providing detailed insights into dislocation propagation and interaction mechanisms. Concurrently, the common neighbor analysis (CNA) method was employed to investigate the formation and development of planar defects, enabling a comprehensive understanding of defect dynamics at the atomic scale. The dislocation density was quantitatively determined by calculating the total length of dislocation lines per unit volume, ensuring an accurate representation of the material’s defect state. Strain was calculated using custom implementation within the LAMMPS 3 Mar 2020 simulation package.

### 2.1. Slip Trace Analysis Method

The SEM observation of the polished surface of the tensile samples successfully captured various slip activities during the in situ tensile process. To identify the active slip systems at each loading step, Schmid factor (SF) calculations and slip trace analyses were performed based on the results obtained from SEM and EBSD observations.

According to Schmid’s law [18,19], the slip system with the highest Schmid factor (SF) value is typically activated (the deformation compatibility between adjacent grains may force the activation of slip systems with low SF). Therefore, the first step in identifying slip activity is to calculate the SF values for all considered slip systems. Subsequently, the slip system with the highest theoretical SF value is identified and assumed to be the active slip system. The calculation of SF values involves three primary steps: extracting the Euler angles (ϕ1, ϕ, ϕ2) from the EBSD data to determine the crystallographic orientation of the grain; transforming the tensile direction (T) into the crystal coordinate system; and calculating the SF values.

The coordinate transformation matrix, g, is defined based on the Euler angles, and the formula is given as:

(1) $$g=\begin{array}{ccc} \cos\phi 1\cos\phi 2-\sin\phi 1\sin\phi 2\cos\phi & \sin\phi 1\cos\phi 2+\cos\phi 1\sin\phi 2\cos\phi & \sin\phi 2\sin\phi \\ -\cos\phi 1\sin\phi 2-\sin\phi 1\cos\phi 2\cos\phi & -\sin\phi 1\sin\phi 2+\cos\phi \cos\phi 2\cos\phi & \cos\phi 2\sin\phi \\ \sin\phi 1\sin\phi & -\cos\phi 1\sin\phi & \cos\phi \end{array}$$

Subsequently, the tensile direction vector (T) is transformed into the crystal coordinate system to ensure consistent calculations within the same coordinate framework. In this study, the tensile load is applied along the [100] axis in the sample coordinate system. The new vector of the tensile direction in the crystal coordinate system (Tc) can be calculated as:

(2) $$T_{c}=g\cdot T$$

Therefore, the Schmid factor (SF) for the slip system can be calculated as [5]:

(3) $$\mathrm{SF}=\frac{n\cdot T_{c}}{\left|n\right|\cdot \left|T_{c}\right|}\cdot \frac{s\cdot T_{c}}{\left|s\right|\cdot \left|T_{c}\right|}$$

Here, n represents the normal vector of the slip plane and s denotes the slip direction. The SF values for all equivalent slip systems are calculated using the equation. The slip system with the highest SF value is considered activated.

Slip trace analysis is typically conducted by comparing theoretical traces with experimental observations. A slip trace is defined as the intersection line between the slip plane and the observation surface of the tensile sample. The normal vector of the slip trace ($s_{T}$) is calculated using the following formula:

(4) $$s_{T}=n\times N_{c}$$

Here, n represents the normal vector of the slip plane, while Nc denotes the normal vector of the observation surface in the crystal coordinate system. The vector Nc can be obtained by transforming the normal vector of the observation surface in the sample coordinate system (N) using the transformation matrix g, expressed as Nc = g × N. Additionally, the angle (θ) between the slip trace and the tensile direction is calculated using the following formula [20]:

(5) $$\theta =\arccos\frac{s_{T}\cdot T_{c}}{\left|s_{T}\left|\cdot \right|T_{c}\right|}$$

The active slip system can be determined by comparing the theoretical θ values with the experimental observations. Typically, a deviation tolerance of ±5° is applied when comparing the theoretical and experimental values. However, slip trace analysis may fail to distinguish slip systems with similar traces. Therefore, Schmid’s law is commonly combined with slip trace analysis to accurately identify the activated slip system.

### 2.2. Calculation Method for Geometrical Compatibility Factor (GCF)

The geometrical compatibility factor (m′) is a parameter used to describe the coordination of slip system activation between adjacent grains and is commonly employed to analyze the plastic deformation behavior of polycrystalline materials. It reflects the degree of geometrical alignment between the slip systems in neighboring grains. A higher m′ value indicates better coordination between slip systems, facilitating easier slip transfer. The value of m′ ranges from 0 to 1, where 0 signifies that slip is completely hindered by the grain boundary and 1 indicates that the grain boundary poses no obstruction. The formula for calculating m′ is as follows [21]:

(6) $$m^{\prime }=\cos\psi \cdot \cos\kappa$$

Here, ψ represents the angle between the normal vectors of the slip planes on either side of the grain boundary, while κ denotes the angle between the slip direction vectors in neighboring grains. Previous studies have shown that slip transfer can occur under two necessary conditions. First, the activated slip systems in the two adjacent grains must exhibit high Schmid factor (SF) values, indicating the potential slip systems that are likely to be activated under tensile loading. Second, the geometrical compatibility factor (m′) between the adjacent grains must exceed 0.7, suggesting a high degree of geometrical compatibility between the grains, Multiple slip systems are often activated during actual deformation. However, m′ is calculated for a single slip system pair and cannot evaluate the influence of multi-slip system interactions on slip transfer. However, in actual deformation, multiple slip systems are frequently activated. Since m′ is calculated for a single slip system pair, it cannot evaluate the influence of interactions between multiple slip systems on slip transfer [22,23].

## 3. Results

**Figure 1:**
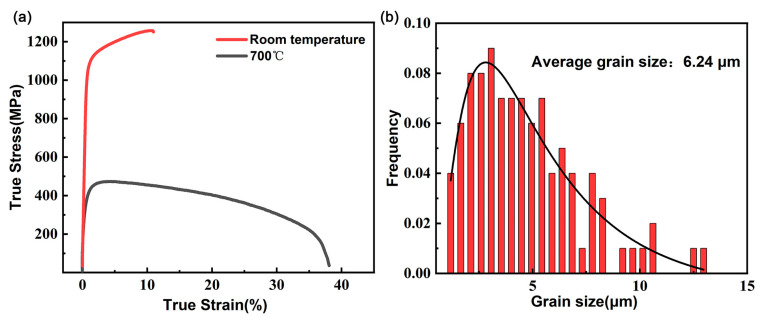
(**a**) True stress–strain curves for tensile tests conducted at room temperature and 700 °C. (**b**) Histogram showing the grain size distribution and the average grain size.

The tensile true stress–strain curves of Ti65 alloy, shown in Figure 1, demonstrate its distinct mechanical responses at different temperatures. At room temperature, the alloy exhibits remarkable strength with an ultimate tensile strength (UTS) of 1144 MPa, while maintaining a total elongation of 10.0%. However, when tested at 700 °C, the material shows dramatically different behavior: the UTS decreases substantially to 456 MPa (a 60% reduction), but the plasticity improves remarkably to 45% elongation. The observed mechanical transition suggests fundamental changes in deformation mechanisms between room temperature and high-temperature conditions.

**Figure 2:**
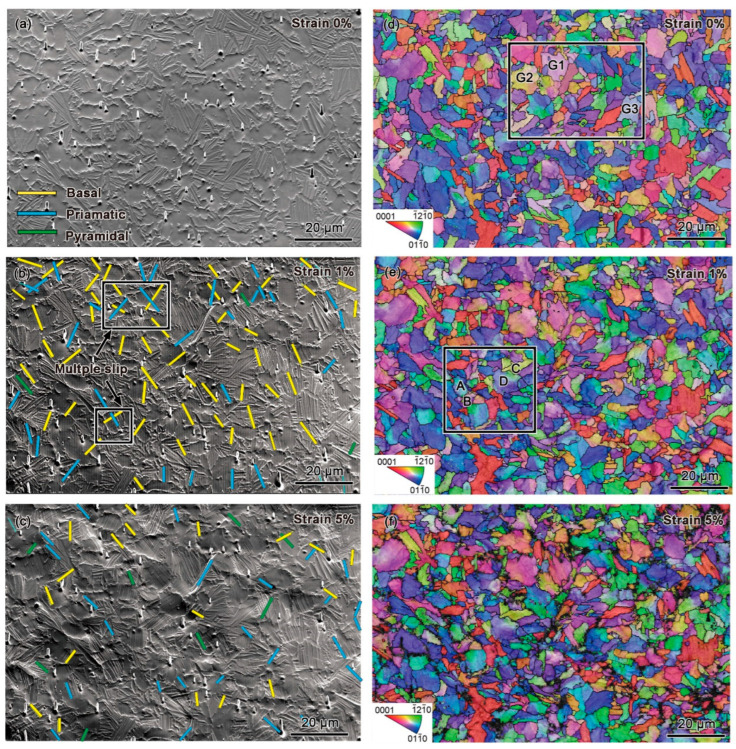
In situ tensile testing of Ti65 alloy at room temperature: (**a**–**c**) SEM images and slip trace analyses at 0%, 1%, and 5% strain, respectively; (**d**–**f**) IPF maps at 0%, 1%, and 5% strain, respectively.

Compared to room-temperature tensile tests, the high-temperature tensile curves display longer steady-state flow stages accompanied by lower stress levels, as shown in Figure 1a. Figure 2a,d illustrate that the microstructure of the in situ specimens primarily consists of equiaxed primary α phase and lamellar secondary α phase, with an average grain size of 6.24 µm and a relatively wide grain size distribution, and the coefficient of determination (R^2^) is 0.908.

Slip occurs through the motion of dislocations, which glide on the crystal’s slip planes, resulting in relative displacement of the crystal structure. Generally, the greater the number of activated slip systems in an alloy, the better its plasticity. Therefore, the types of slip traces observed in SEM images, which are associated with dislocation slip, can be identified by combining them with crystallographic orientation data [16]. The slip trace method has been widely applied to determine deformation mechanisms and crack propagation directions [24].

**Figure 3:**
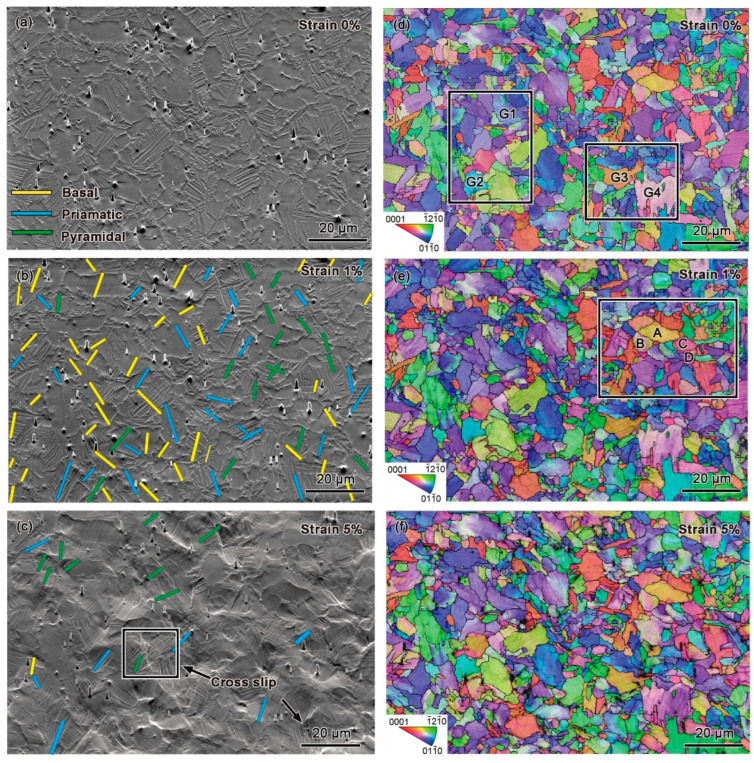
In situ tensile testing of Ti65 alloy at 700 °C: (**a**–**c**) SEM images at 0%, 1%, and 5% strain, respectively; (**d**–**f**) IPF maps at 0%, 1%, and 5% strain, respectively.

To identify the activated slip systems of Ti65 alloy at room temperature and 700 °C, detailed slip trace analyses were conducted on the surfaces of tensile-deformed samples. Figure 2 and Figure 3 show the results of slip trace analyses and corresponding inverse pole figure (IPF) maps for the same location at different strain levels during in situ tensile testing of Ti65 alloy at room temperature and 700 °C, respectively. In these figures, the yellow, blue, and green lines represent basal, prismatic, pyramidal <a>, and pyramidal <c+a> slip systems activated in the corresponding grains.

Figure 2a shows the initial microstructure of the alloy under room-temperature tension, revealing undeformed primary grains with no slip lines present. In Figure 2b, the slip trace analysis for room temperature tension at a strain of 1% indicates that slip traces were observed in 129 grains (where grains with the same orientation within lamellae were considered a single grain). At this strain level, deformation under room-temperature tension was primarily dominated by basal and prismatic slip, with only a small fraction of pyramidal slip systems activated. Additionally, nine grains exhibiting multi-slip were identified, all of which were equiaxed α grains. Multi-slip refers to the activation of multiple slip systems in different directions within the same grain. This phenomenon commonly occurs in grains with multiple available slip planes, particularly in equiaxed primary α grains where the stress distribution is relatively uniform.

Multi-slip contributes to more uniform deformation within grains, reducing localized deformation caused by single-slip-dominated mechanisms. It has been well established that multi-slip promotes dislocation entanglement, reduces the average dislocation mean free path, and consequently increases flow stress. As the strain increased to 5%, as shown in Figure 2c, slip activity continued in 50 grains within the observed region. The proportion of prismatic and basal slip decreased, while the proportion of pyramidal slip increased. Only two instances of multi-slip were observed, and no evidence of cross-slip was detected.

Figure 3b shows the slip trace analysis of Ti65 alloy during high-temperature tensile testing at 700 °C with a strain of 1%. A total of 98 grains exhibiting slip traces were analyzed. At a strain of 1%, compared to room-temperature tension, pyramidal slip was observed to be significantly more prevalent under high-temperature tension, while the proportions of basal and prismatic slip decreased. Additionally, two grains were identified as exhibiting multi-slip.

As the strain increased to 5%, as shown in Figure 3c, slip activity continued in 17 grains within the observed region. The proportion of prismatic and basal slip further decreased, while the proportion of pyramidal slip increased. Furthermore, multiple instances of stepped cross-slip were observed. Cross-slip typically occurs when dislocations moving on one slip plane encounter obstacles (such as phase interfaces, grain boundaries, or regions of high dislocation density) and are forced to transfer to another slip plane. This phenomenon is more commonly observed in irregularly shaped grains or structures with pronounced internal stress concentrations, such as lamellar grains within the secondary α phase. Due to the high aspect ratio of the secondary α phase and the presence of numerous grain boundaries within the same colony, dislocations frequently encounter obstacles during slip, providing favorable conditions for cross-slip.

**Figure 4:**
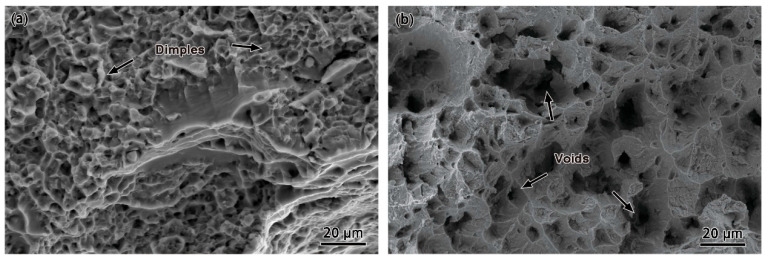
Ti65 alloy: (**a**) Fracture surface from room-temperature tensile testing; (**b**) Fracture surface from high-temperature tensile testing.

To elucidate the difference between the high-temperature and room-temperature tensile properties of Ti65 alloy, the tensile fracture morphology of the alloy at the two temperatures was analyzed by comparison in Figure 4. The room-temperature tensile fracture is mainly characterized by a high density of small shallow dimples, and obvious tear edges and dimple belts can be observed in local areas. The fracture morphology shows that there are microporous aggregation-type toughness fracture and local brittle fracture characteristics during the fracture process, which presents a typical tough–brittle mixed fracture mode. In contrast, the fracture of the high-temperature tensile specimen shows the trajectories of microvoid aggregation extending in different directions. The microstructure is dominated by tiny uniformly distributed equiaxed dimples, and further analysis shows that the material exhibits more significant toughness fracture characteristics under high-temperature conditions. In a recent study, Pilchak and Williams [25] argued that the notch features in near-α titanium alloys are actually anisotropically grown microvoids in the primary α phase because the hexagonal crystal structure is less symmetric than the cubic structure. They suggested that, when the primary α grains are oriented to slip easily on prismatic planes, the notches nucleate at the intersection of the slip zone and grain boundaries, and these notches are connected to the primary cracks and thus fracture occurs. He et al. [26,27] noted that, in Ti60 titanium alloy, cracks predominantly initiate from subsurface regions, particularly at α/β phase boundaries or α grain boundaries. The higher hardness of the α phase and the greater toughness of the β phase lead to a mismatch in Young’s modulus at the phase interfaces, resulting in localized stress concentrations. These stress concentrations promote the formation of cleavage facets, which serve as crack initiation sites.

## 4. Discussion

### 4.1. Slip Trace Analysis

For near-α or α titanium alloys, the low symmetry of the HCP crystal structure leads to complex microdeformation mechanisms. Typically, three types of slip systems are present in these materials, with slip directions characterized by <a>-type Burgers vectors. These systems operate on the basal planes {0001}, prismatic planes {10$\bar{1}$0}, and first-order pyramidal planes {10$\bar{1}$1}. According to the Taylor–von Mises criterion, five independent slip systems are required to accommodate arbitrary grain shape changes during plastic deformation [28]. However, these three <a>-type slip systems are insufficient to meet this requirement. Therefore, additional deformation mechanisms, such as pyramidal <c+a> slip or twinning, must be activated to maintain the plastic deformation compatibility in polycrystals.

**Figure 5:**
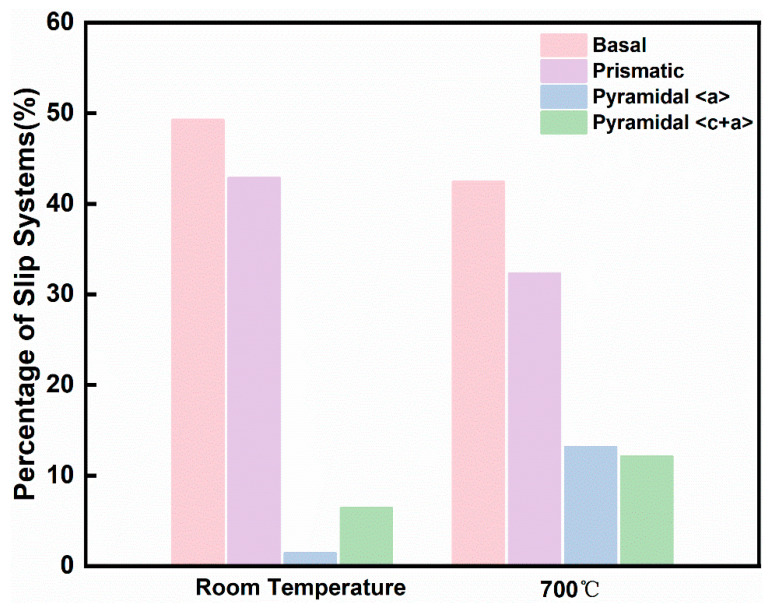
Relative frequency of activated slip modes observed in Ti65 alloy at 5% strain during tensile testing at room temperature and 700 °C.

Figure 5 illustrates the percentage of different slip systems activated at a strain of 5% during tensile testing of Ti65 alloy at room temperature and 700 °C. As shown in the figure, at room temperature, basal slip, prismatic slip, pyramidal <a> slip, and pyramidal <c+a> slip were observed. During this stage of plastic deformation, basal slip and prismatic slip are the dominant mechanisms, while pyramidal <a+c> slip is rarely activated and essentially does not contribute to the deformation. Slip activity is not only dependent on the crystallographic orientation but also on the critical resolved shear stress (CRSS) of each slip system [29]. Prismatic and basal slip are considered the easiest to activate due to their relatively low CRSS, whereas pyramidal slip and deformation twinning typically require higher applied stress. For most near-α titanium alloys, prismatic slip is the preferentially activated system [30]. In addition to prismatic slip, the slip system involving the (0001) plane and [11–20] direction is another system that is relatively easy to activate.

In contrast, during high-temperature tensile testing at 700 °C, the activation proportions of basal and prismatic slip were significantly reduced, while a large number of pyramidal <a> slip and pyramidal <c+a> slip systems were activated. The proportion of pyramidal <c+a> slip among the statistically analyzed slip traces increased from 6.4% to 12.1%, while the proportion of pyramidal <a> slip increased from 1.4% to 13.1%. Only a small amount of multiple slips were observed. The contribution of <c+a> dislocations and their cross-slip to ductility can be discussed from two aspects. First, as indicated by the Taylor–von Mises criterion, <c+a> dislocations can accommodate plastic strain along the c-axis, promoting stable plastic flow. Second, the pronounced tendency of <c+a> dislocations to cross-slip between Pyramidal-I and Pyramidal-II planes further contributes to deformation homogenization, thereby enhancing ductility. The study by H. Li et al. [31] revealed that the relative activity of different deformation systems varies with alloy composition and deformation temperature, and the increase in temperature significantly reduces the CRSS required to activate pyramidal slip.

Additionally, as the tensile strain increases, microstructural observations of the deformed material reveal the presence of microcracks. These microcracks are predominantly concentrated within slip bands inside the grains, primarily caused by the intense strain mismatch that induces stress cracking. These microcracks can quickly coalesce to form major cracks [32], which eventually propagate with the significant increase in tensile loading, leading to the fracture of the tensile specimen.

### 4.2. Coordinated Deformation Mechanisms

#### 4.2.1. Crystal Orientation Rotation

To accommodate the increased macroscopic strain, a large number of slip systems must be activated during plastic deformation. Therefore, to facilitate slip activation, the crystallographic orientation of grains typically undergoes a certain degree of rotation [33,34]. Crystal orientation rotation is one of the mechanisms through which deformation is accommodated in alloys, and it is often associated with dislocation motion, particularly dislocation slip. Figure 2 and Figure 3 illustrate the overall deformation behavior of all grains in Ti65 alloy during tensile testing at room temperature and at 700 °C. However, examining the details of orientation changes in individual grains reveals that orientation rotation is non-uniform not only between different grains but also within a single grain.

**Figure 6:**
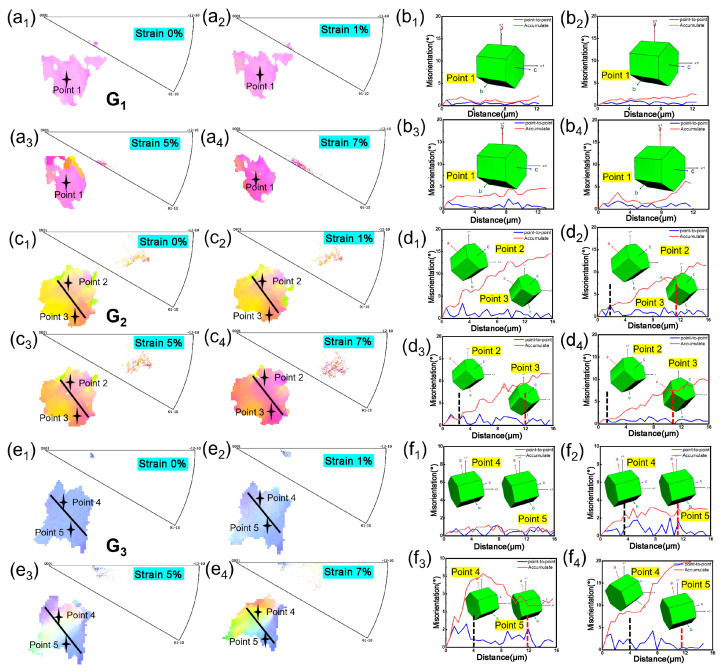
Crystal orientation evolution of selected grains in Ti65 alloy during in situ tensile testing at room temperature: (**a1**–**a4**,**c1**–**c4**,**e1**–**e4**) Evolution of crystal orientation with increasing global strain (0%, 1%, 5%, 7%) and corresponding inverse pole figure (IPF) maps; (**b1**–**b4**,**d1**–**d4**,**f1**–**f4**) Orientation distribution analysis along specific lines within the grains and the lattice unit at specific points in corresponding strain states (0%, 1%, 5%, and 7%).

**Figure 7:**
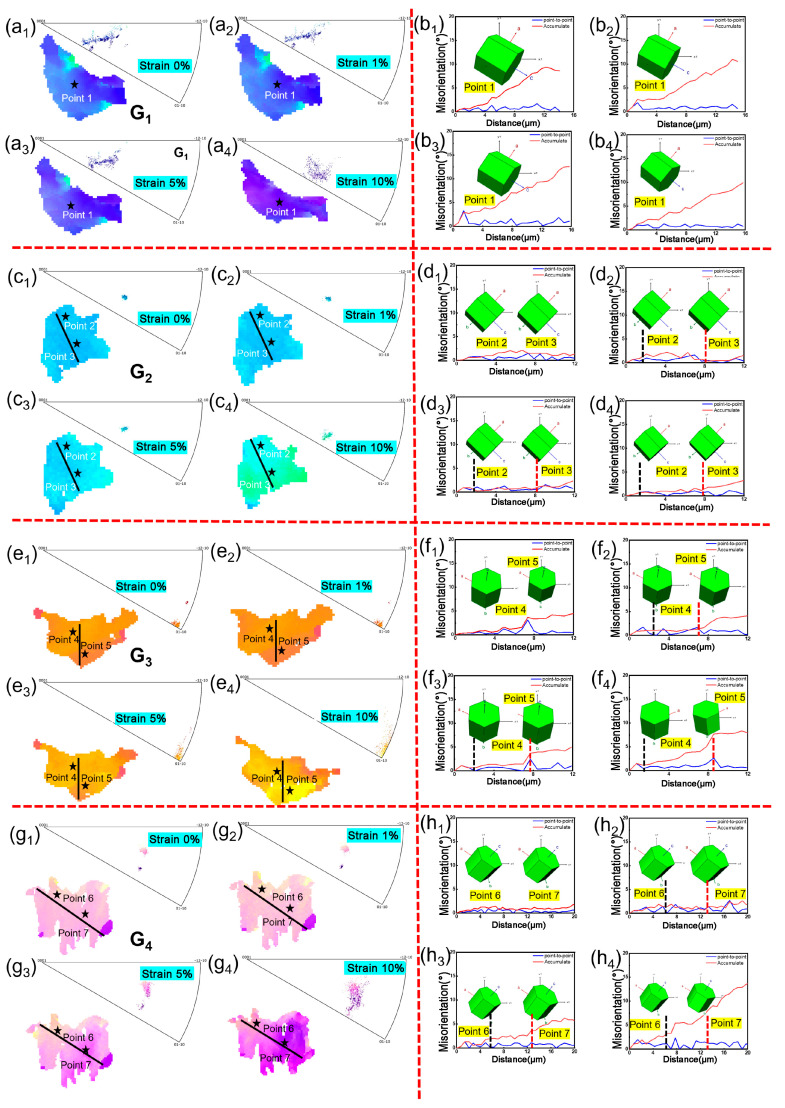
Crystal orientation evolution of selected grains in Ti65 alloy during in situ tensile testing at 700 °C: (**a1**–**a4**,**c1**–**c4**,**e1**–**e4**,**g1**–**g4**) Evolution of crystal orientation with increasing global strain (0%, 1%, 5%, 10%) and corresponding inverse pole figure (IPF) maps; (**b1**–**b4**,**d1**–**d4**,**f1**–**f4**,**h1**–**h4**) Orientation distribution analysis along specific lines within the grains and the lattice unit at specific points in corresponding strain states (0%, 1%, 5%, and 10%).

Figure 6 and Figure 7 present the grain orientation rotation information for room-temperature and 700 °C high-temperature tensile tests, respectively. The regions containing the selected grains are indicated by the white dashed boxes in Figure 2 and Figure 3d, respectively. It can be observed that, during tensile deformation, the IPF color of grains changes with increasing strain, demonstrating that tensile strain induces grain orientation rotation. Additionally, the linear distribution of orientation differences along specific marked lines within grains was plotted to reveal the orientation rotation of individual grains. Before deformation, the linear distribution was relatively uniform, indicating a unique orientation for the grain. After deformation, fluctuations in the curve signify grain rotation, with an increase in relative orientation differences.

Significant differences were observed in grain orientation rotation between different tensile temperatures. During room-temperature tensile testing, fewer grains exhibited orientation rotation, but the magnitude of orientation rotation varied significantly between different grains. Figure 6(a1–a4,c1–c4,e1–e4) show the evolution of selected grains with increasing strain, while Figure 6(b1–bd,d1–d4,f1–f4) display the orientation distribution along specific lines within grains and the lattice unit at particular points. Grain G1 exhibited an overall trend of rotation with increasing strain, but the orientation change was minor, with small directional deviations. In contrast, grains G2 and G3 displayed localized rotation within different regions of the same grain, with distinct differences in color within the grain. For grain G3, the maximum internal orientation deviation spanned approximately 20°. Moreover, different regions within the same grain exhibited varying lattice rotation directions, and the degree of orientation rotation differed significantly between grains, indicating localized heterogeneity in plastic deformation. Throughout the tensile deformation process, the points on the inverse pole figure changed from a clustered state to a scattered state, indicating that the orientation within individual grains gradually became non-uniform. Different regions within the same grain exhibited varying lattice rotation directions, and the degree of orientation rotation differed significantly between grains, indicating localized heterogeneity in plastic deformation. Furthermore, as clearly seen in the IPF maps of the crystals in Figure 6(a1–a4,c1–c4,e1–e4) the lattice distortion near the grain boundaries within the same grain is more severe relative to the grain interior. The severe stress concentration at the grain boundaries, which cannot be released, can lead to the initiation of microcracks and subsequently result in fracture.

Compared to the evolution of grain orientations at room temperature, a larger number of grains exhibited orientation rotation during high-temperature tensile testing. However, as shown in Figure 7, the overall color change in the IPF map at 700 °C was smaller, and the orientation differences were less pronounced. This suggests that grain orientations underwent limited rotation at high temperatures. Further analysis of the non-uniformity in internal grain orientation evolution, as indicated by the marked positions within the grains, revealed that the orientation deviation within all grains remained within approximately 5°. Points in the pole figure remained almost clustered throughout the tensile deformation process, indicating relatively uniform orientation within individual grains. Figure 7(a1–a4,c1–c4,e1–e4,g1–g4) show the evolution of selected grains with increasing strain during high-temperature tensile testing, while Figure 7(b1–b4,d1–d4,f1–f4,h1–h4) display the orientation distribution along specific lines within grains and the lattice unit at particular points during high-temperature tensile testing. Compared to the evolution of grain orientations at room temperature, a larger number of grains exhibited orientation rotation during high-temperature tensile testing. Unlike the results from room-temperature tensile testing in Figure 6, as shown in Figure 7, the overall color change in the IPF map at 700 °C was smaller, and the orientation differences were less pronounced. This suggests that grain orientations underwent limited rotation at high temperatures. Furthermore, as shown in Figure 7, for grains G1–G3, the grain color changed uniformly overall with increasing strain, and no significant stress concentration existed at the grain boundaries. Only a small number of grains, such as G4, exhibited varying lattice rotation directions in different regions within the same grain.

These findings suggest that, during the plastic deformation, grain orientation rotation plays an important role in promoting dislocation slip and providing coordinated deformation mechanisms. The limited grain orientation rotation and the uniformity of internal grain orientations during high-temperature tensile testing may be related to the coordinated effects of grain boundary sliding and dislocation motion at high temperatures. At high temperatures, grain boundary sliding may become the dominant deformation mechanism, effectively coordinating deformation between grains and thus reducing local orientation changes within grains.

#### 4.2.2. Grain Boundary Slip Transfer

As described above, in addition to grain orientation rotation, coordinated deformation between adjacent grains becomes necessary to accommodate the increasing strain during deformation, particularly under high-temperature tensile conditions. Typically, such intergranular coordinated deformation is achieved through grain boundary slip transfer [35]. Therefore, the m′ is employed to quantitatively describe slip transfer between adjacent grains.

Furthermore, slip transfer can effectively coordinate the increased macroscopic strain and relieve local stress concentrations at the GB. If the slip systems on either side of the GB are misaligned and the m′ value is very low, slip transfer across the GB becomes difficult. Dislocations accumulate near the GB, increasing the density of geometrically necessary dislocations (GNDs). These GNDs cause localized lattice rotation, which can be quantified using the kernel average misorientation (KAM). An uneven distribution of KAM values indicates the presence of pronounced local deformation [36,37].

**Figure 8:**
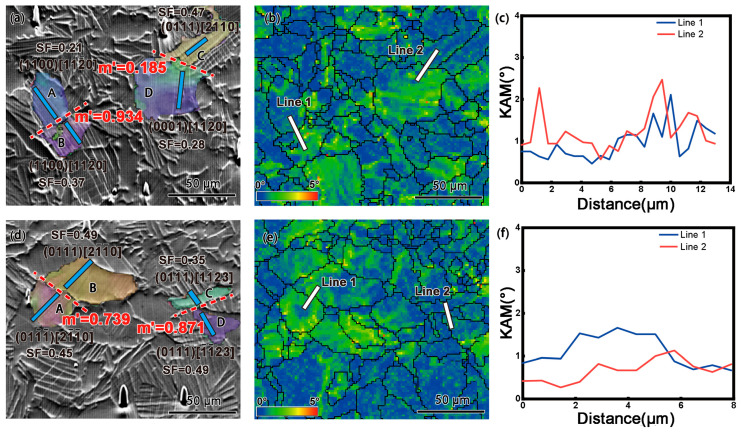
Slip transfer behavior between two sets of selected grains in Ti65 alloy during tensile testing at room temperature (**a**–**c**) and high temperature (**d**–**f**): (**a**,**d**) IPF + SEM images; (**b**,**e**) KAM maps; (**c**,**f**) Linear distribution of KAM values.

Figure 8a–c illustrate slip transfer between two sets of adjacent grains under room-temperature tensile conditions, and the selected region is within the white dashed box in Figure 2e. In grain A, the slip bands cross the grain boundary and enter grain B. Based on EBSD data and SEM observations, the Schmid factor (SF) values and m′ of the slip systems were calculated, as shown in Figure 8a. It was found that prismatic slip systems were activated in grains A and B, with SF values of 0.21 and 0.37, respectively. The m′ value between grains A and B was 0.934, significantly higher than 0.7, indicating high geometrical compatibility and the occurrence of prismatic slip transfer between grains A and B. However, the m′ value between grains C and D was 0.185, far below 0.7, indicating poor geometrical compatibility and the absence of slip transfer. Figure 8b shows the KAM map of the region, while Figure 8c displays the linear distribution of KAM values perpendicular to the A/B and C/D grain boundaries. It can be observed that significant strain accumulates at the non-slip-transferred C/D boundary.

Figure 8d–f depict slip transfer between two sets of adjacent grains under high-temperature tensile conditions, and the selected region is within the white dashed box in Figure 3e (The blue dashed line indicates the direction of its slip trace). Compared to room-temperature slip transfer, a larger number of grains exhibited slip transfer at high temperatures. The m′ values between grains A/B and C/D were 0.739 and 0.871, respectively, both exceeding 0.7, indicating high geometrical compatibility and effective prismatic slip transfer between the two sets of grains. Moreover, the activation of non-basal slip systems at high temperatures further increased the likelihood of slip transfer, diversifying the slip transfer pathways. By comparing KAM values, it was also found that the KAM values at the grain boundaries of the two sets of grains were lower under high-temperature tensile conditions than at room temperature, with a more uniform strain distribution.

Overall, the slip transfer mechanism at high temperatures exhibits greater efficiency and coordination. Grain boundary slip transfer effectively coordinates deformation between adjacent grains, reduces local stress concentrations at grain boundaries, and enhances the overall plastic deformation capacity of the material. These phenomena and discussions elucidate the temperature-dependent deformation mechanisms of the Ti65 alloy, providing critical insights into its high-temperature mechanical behavior. These findings offer valuable guidance for optimizing thermomechanical processing, enhancing high-temperature mechanical properties, and expanding its engineering applications.

#### 4.2.3. Slip Modes and Microstructural Evolutions

**Figure 9:**
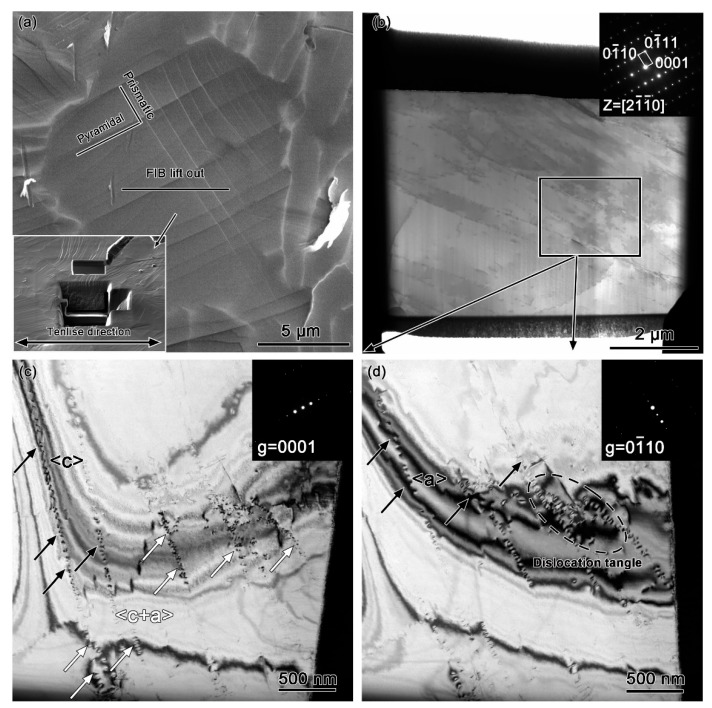
TEM image of equiaxed grains exhibiting pyramidal and prismatic slip under 15% strain at high temperatures. (**a**) FIB-TEM sampling location; (**b**) [2–1–10] zone axis TEM images of the α-Ti grain (the inset shows the corresponding SAED pattern); (**c**) g = [0001] bright field image showing <c> and <c+a> dislocations; (**d**) g = [01–10] bright field image showing <a> and <c+a> dislocations.

To investigate the mechanisms of grain orientation rotation at high temperatures, TEM specimens were extracted via FIB from equiaxed grains exhibiting pyramidal and prismatic slip under 15% strain, with the sampling location illustrated in Figure 9a. TEM analysis was performed under two-beam diffraction conditions using specific diffraction vectors to characterize deformation modes.

As shown in Figure 9c under the g = [0002] diffraction vector, two distinct dislocation types were observed. According to the g·b = 0 invisibility criterion (where g is the diffraction vector and b the Burgers vector), these correspond to <c> (black arrows) and <c+a> dislocations (white arrows). The g·b × u = 0 criterion (u representing dislocation line direction) further identifies these as prismatic <c> edge dislocations (b = [0001]) and pyramidal <c+a> edge dislocations (b = 1/3[11–23]).

When using the g = [01–10] diffraction vector (Figure 9d), <a> (black arrows) and <c+a> dislocations became visible while <c> dislocations remained invisible. Notably, extensive dislocation tangles within white dashed boxes were observed forming low-angle grain boundaries (LAGBs). These microstructural features develop through coordinated activation of numerous pyramidal <c+a> dislocations to accommodate substantial c-axis strain. The dislocation tangles progressively organize into energy-reducing dislocation walls, which subsequently evolve into LAGBs through dislocation annihilation and rearrangement processes.

This evidence demonstrates that pyramidal <c+a> slip dominates plastic deformation in these grains. The localized grain rotation observed in Figure 9 originates from dislocation tangle networks containing pyramidal <c+a> dislocations, which collectively coordinate plastic deformation through this unique orientation adjustment mechanism. The transition from random dislocation tangles to organized LAGBs represents a critical microstructure evolution pathway for stress accommodation under high-temperature deformation conditions.

#### 4.2.4. MD Analysis of Dislocation During Tensile Simulations

**Figure 10:**
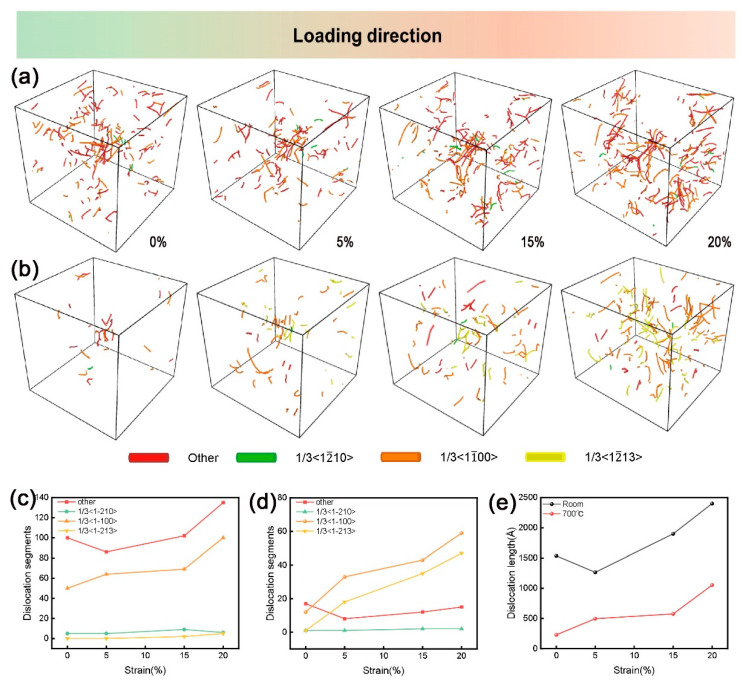
(**a**) Distribution of different dislocations during tensile deformation at room temperature and (**b**) 700 °C, where red indicates other types of dislocations, green represents 1/3<1–210> dislocations, orange denotes 1/3<1–100> dislocations, and yellow signifies 1/3<11–23> dislocations; (**c**) The number of different dislocation types during tensile deformation at room temperature and (**d**) 700 °C; (**e**) a comparison of the total length of dislocation lines at room temperature and 700 °C.

Finally, MD was used to conduct tensile simulations of the polycrystalline model at room temperature and 700 °C, providing more valuable insights for the conclusion of this paper through atomic-scale simulation calculations. Figure 10 shows the dislocation characteristics of the polycrystalline model obtained from molecular dynamics simulation at different strain stages at room temperature and 700 °C and quantitatively calculates the number of various dislocations and the total dislocation line length. The dislocation density is the total dislocation line length divided by the model volume. Therefore, the dislocation density is directly proportional to the total dislocation line length. At room temperature (273 K), during the tensile deformation process, the Shockley incomplete dislocation with 1/3<1–100> Burgers vector was mainly activated (Figure 10a). This type of dislocation has relatively low energy and is an important carrier for plastic deformation in closely arranged hexagonal (HCP) Ti. In addition, other types of dislocations (such as total dislocations) were also observed. The number and total length of these dislocations gradually increase with the increase in strain (Figure 10c,e), indicating that dislocation proliferation and accumulation are the main mechanisms of room-temperature plastic deformation.

The total dislocation line length at high temperature was significantly lower than that at room temperature (Figure 10e). This phenomenon is attributed to the dislocation recovery and possible recrystallization process that occurs at a high temperature of 700 °C. High temperature promotes the climbing, annihilation, and rearrangement of dislocations, resulting in the elimination of dislocation substructures and the reduction of internal stress. This leads to the fact that, although dislocations are constantly generating and moving, their net accumulation rate decreases, and the total length reduces. It reflects the enhanced thermal activation effect of the dislocation annihilation process at high temperatures, enabling the material to maintain a relatively low dislocation storage level during the deformation process.

The 1/3<1–100> incomplete dislocations observed at room temperature are usually produced by the dissociation of the <a> type total dislocations with 1/3<1–210> Burgers vectors on a specific sliding plane, and the reaction equation is:

1/3<1–210> → 1/3<1–100> + 1/3<0–110>

Because 1/3<1–210> dislocations have a relatively low critical shear stress (CRSS), they are usually the first dislocations in HCP titanium to be activated to adapt to plastic deformation. The incomplete dislocations formed by its decomposition are the main types of dislocations observed in room-temperature deformation.

Molecular dynamics simulations clearly reveal the core impact of temperature increase on dislocation activation behavior: thermal activation significantly reduces the CRSS of the originally difficult-to-activate <c+a> dislocations (1/3<11–23>), enabling them to be activated in large quantities at high temperatures (Figure 10b,d). This is highly consistent with the experimental phenomenon that the rheological stress of the material decreases and its plasticity significantly increases at high temperatures (Figure 1a). The simulation results directly support the conclusions of TEM observation (Figure 9) and macroscopic mechanical property tests, jointly clarifying the root cause of the dislocation mechanism for the excellent plasticity of Ti65 alloy at high temperatures.

## 5. Conclusions

The plastic deformation behavior of the Ti65 alloy at room temperature and high temperature was systematically studied through in situ tensile testing. By performing slip trace analysis, crystal orientation analysis, geometrical compatibility factor evaluation, TEM observations, and molecular dynamics simulations, the different deformation mechanisms of Ti65 alloy under these conditions were revealed. Elucidating these mechanisms is crucial for understanding the alloy’s high-temperature mechanical behavior, optimizing thermomechanical processing, and expanding its engineering applicability. Additionally, future research will focus on investigating the dislocation nucleation mechanisms of pyramidal slip in Ti65 alloy at high temperatures.

1. At room temperature, deformation is dominated by basal and prismatic slip with limited pyramidal slip activation, while elevated temperatures significantly promote pyramidal <a> and <c+a> slip due to thermal activation effects, enhancing plasticity. At 5% strain, the proportion of pyramidal <c+a> slip among the statistically analyzed slip traces increased from 6.4% to 12.1%, while that of pyramidal <a> slip increased from 1.4% to 13.1%.
2. Coordinated deformation occurs through crystal rotation and slip transfer—room-temperature deformation features multi-slip and grain rotation to accommodate strain, whereas high-temperature deformation is governed by efficient slip transfer across grain boundaries facilitated by improved geometrical compatibility. This transition in deformation mechanisms explains the alloy’s remarkable plasticity improvement at high temperatures while maintaining moderate strength.
3. TEM observations and molecular dynamics simulations reveal that plastic deformation is predominantly accommodated by basal and prismatic slip systems with minimal pyramidal slip contribution at room temperature. However, thermal activation at elevated temperatures reduces the critical resolved shear stress (CRSS), preferentially activating 1/3<11–23> dislocations and thereby substantially improving plastic deformation capability.

## Data Availability

The original contributions presented in this study are included in the article. Further inquiries can be directed to the corresponding authors.
